# Intramedullary nailing as a treatment for non-unions of femoral shaft fractures after plating failure: A case series

**DOI:** 10.1016/j.ijscr.2023.107908

**Published:** 2023-01-30

**Authors:** Ihsan Oesman, Dody Kurniawan, Anissa Feby Canintika

**Affiliations:** Department of Orthopaedics and Traumatology, Cipto Mangunkusumo Hospital, Jakarta, Indonesia

**Keywords:** Nailing conversion, Implant failure, Femoral shaft nonunion

## Abstract

**Introduction:**

The management of implant failure in femoral shaft fractures remains a challenging problem for orthopaedic surgeons. This series aim to evaluate the effectiveness of intramedullary (IM) nailing for treating femoral shaft nonunions after implant failure.

**Case presentation:**

Three patients presented with pain after walking on crutch and limping with history of fixation using plate for femoral shaft fracture. Implant removal was then performed with subsequent refixation using intramedullary nailing with A2FN. The Lower Extremity Functional Score and Visual Analogue Score evaluation showed excellent result in these patients.

**Clinical discussion:**

IM nailing is the mainstay of treatment for patients with femoral shaft fractures. This intervention provides support to fractures and aid in union of fractures. Several advantages have been reported in IM nailing, including shorter length of stay, rapid union, and early functional capacity of the limb. Insertion of IM nailing may preserve anatomical structure in the patients, which leads to better improvement of functional capacity. Nailing also limits soft tissue damage. Thus, in patients presented with previous plate failure similar to our patients, IM nailing with reaming is recommended.

**Conclusion:**

Nonunion after femoral shaft plating are common. Nailing conversion is one of available treatment options to achieve maximum recover in this type of case.

## Highlights

• The management of implant failure in femoral shaft fracture remains a dilemma for orthopaedic surgeons.
• We treated three patients with aseptic femoral shaft nonunions after implant failure, using intramedullary nailing with A2FN.
• The Lower Extremity Functional Score and Visual Analogue Score evaluation showed excellent result in these patients.

## 1 Introduction and importance

A femoral shaft fracture is a common injury, and the favored treatment nowadays is closed reamed intramedullary (IM) nailing. Despite being commonly used for treating femoral shaft fracture, locked nail is technically demanding with many devices needed to perform a surgery. Therefore, its use may be greatly restricted [1]. In instances where traditional reamed intramedullary nails cannot be used, plates may still be chosen. Previous studies have shown wide range of complication rates when plates were used to treat femoral shaft fractures, with reported nonunion rate of 8 to 19 % [2], [3], [4], [5], [6], [7].

The management of implant failure in femoral shaft fractures, especially if associated with delayed union/non union, remains challenging for orthopaedic surgeons. It represents a serious postoperative complication for the patient, associated with longer time of restricted mobility, shortening, deformity, knee joint stiffness and the need for second major surgery, providing osteosynthesis plus bone graft after failed plate removal [8]. Intramedullary nailing is the gold standard for femoral shaft fracture, with good union results and a success rate up to 95 % [9]. The aim of this series is to evaluate the effectiveness of IM nailing for treating aseptic femoral shaft nonunions after failed plating. This report was conducted in accordance with the 2020 SCARE Guideline [10].

## 2 Presentation of cases

**Fig. 1:**
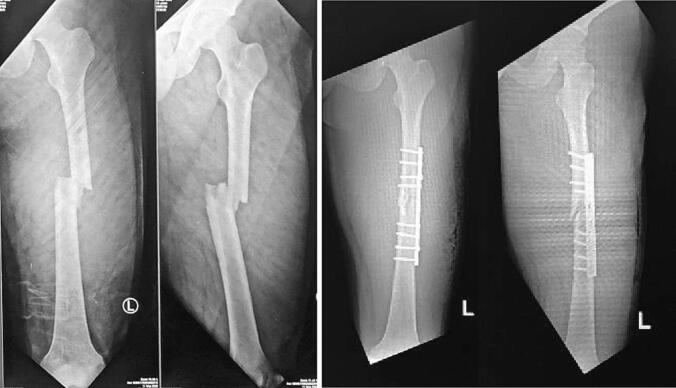
Initial presentation to emergency department and post-operative x-ray with plate fixation.

**Fig. 2:**
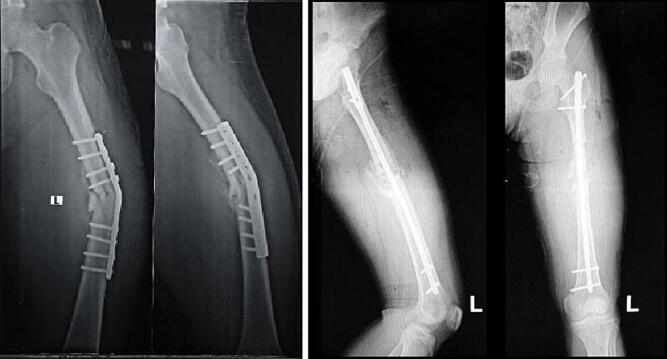
X-ray 2 months after the first surgery showed implant failure. It was then revised with IM nailing.

**Fig. 3:**
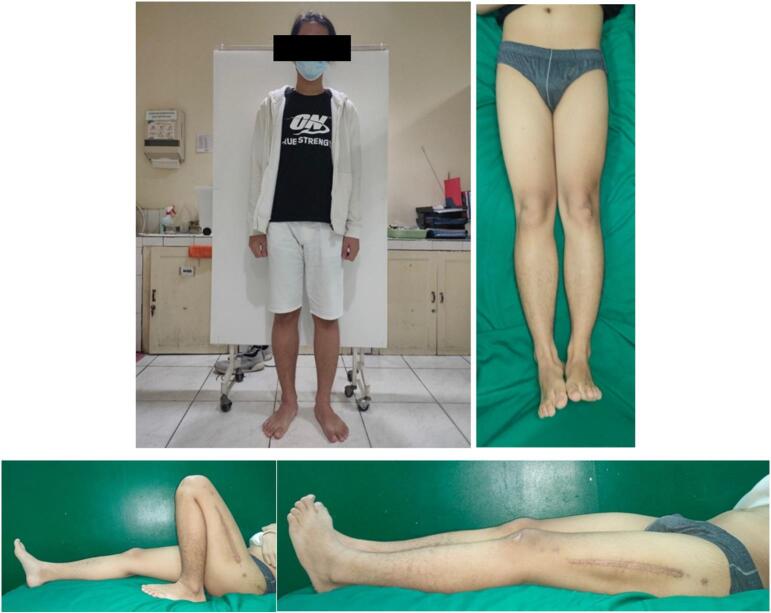
At 6 months of follow up, the patient could already perform full weight bearing and full hip ROM flexion.

**Fig. 4:**
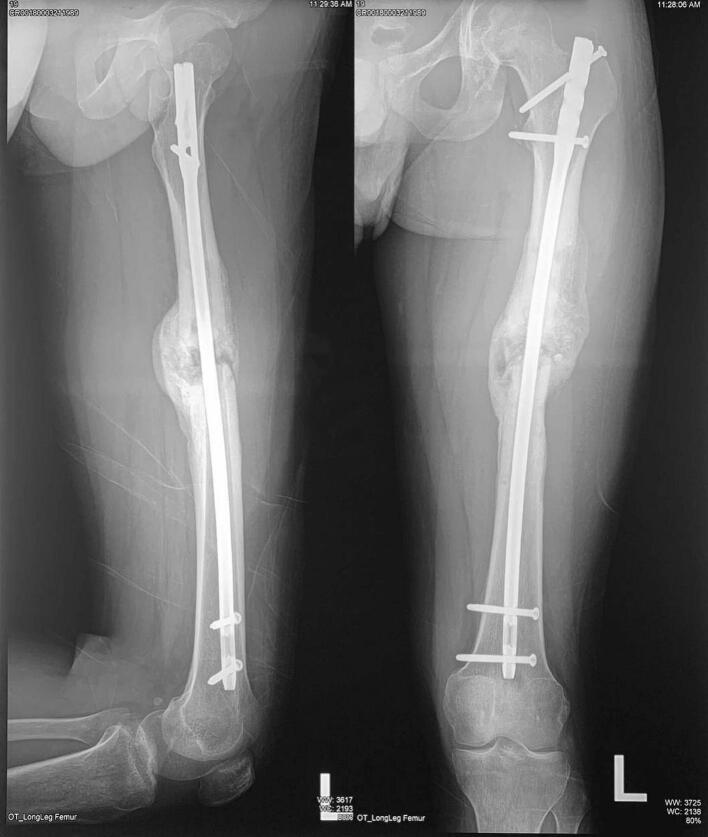
Radiological evaluation after 6 months.

The first case is an 18 year-old-male with closed fracture of the left femoral shaft. The patient underwent ORIF plate and screw in the operating theatre of the emergency department (Fig. 1). The patient was a heavy smoker. Fig. 2 shows the implant failed after 2 months and the patient underwent implant removal and ORIF A2FN. Postoperative x-ray showed satisfying result. The patient was followed-up for 6 months and presented with good clinical and radiological outcome. There was no limitation of range of motion and the patient can mobilize without any difficulties. At six months of follow up, the patient could already perform weight bearing and there was no limitation of range of motion (Fig. 3). Six months after the surgery, x-ray demonstrated partial union (Fig. 4).

**Fig. 5:**
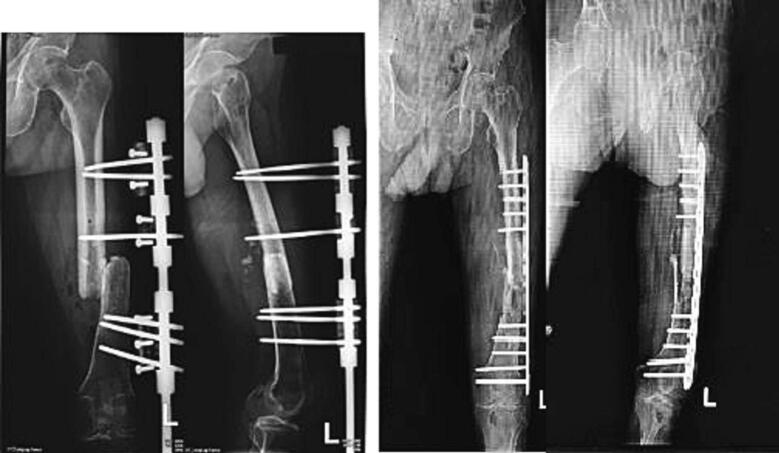
Distraction of nonunion femoral shaft and second surgery by plating.

**Fig. 6:**
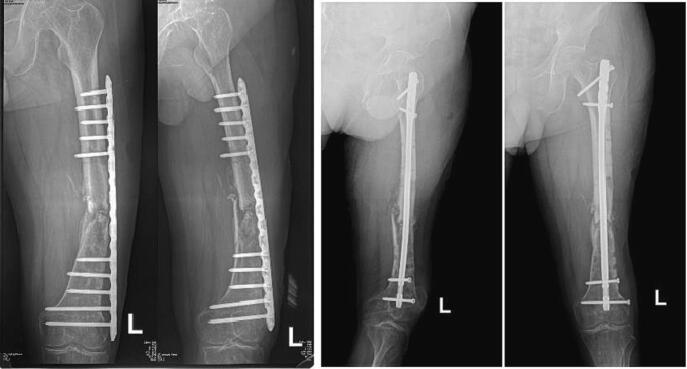
Revision surgery with IM nail following nonunion femoral shaft after fixation with plate.

**Fig. 7:**
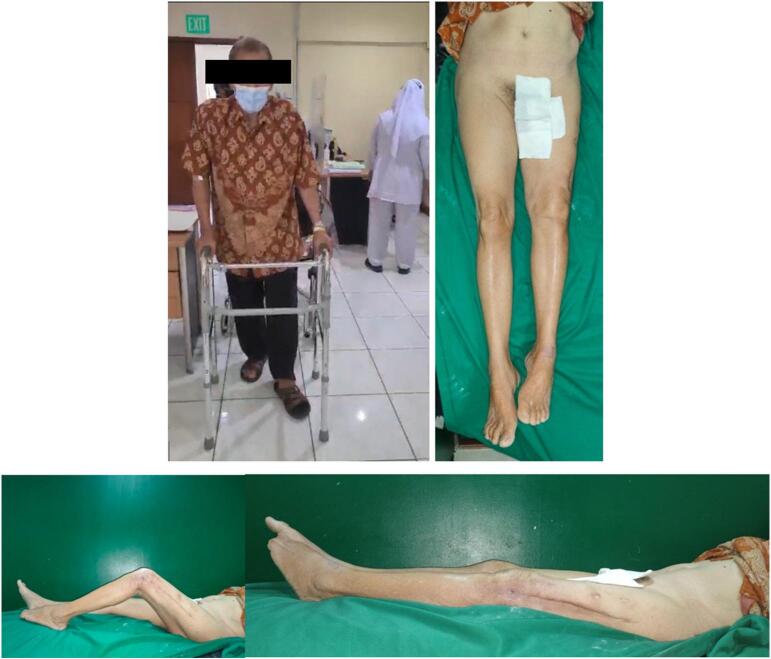
Follow-up after 6 months following revision surgery with IM nail.

**Fig. 8:**
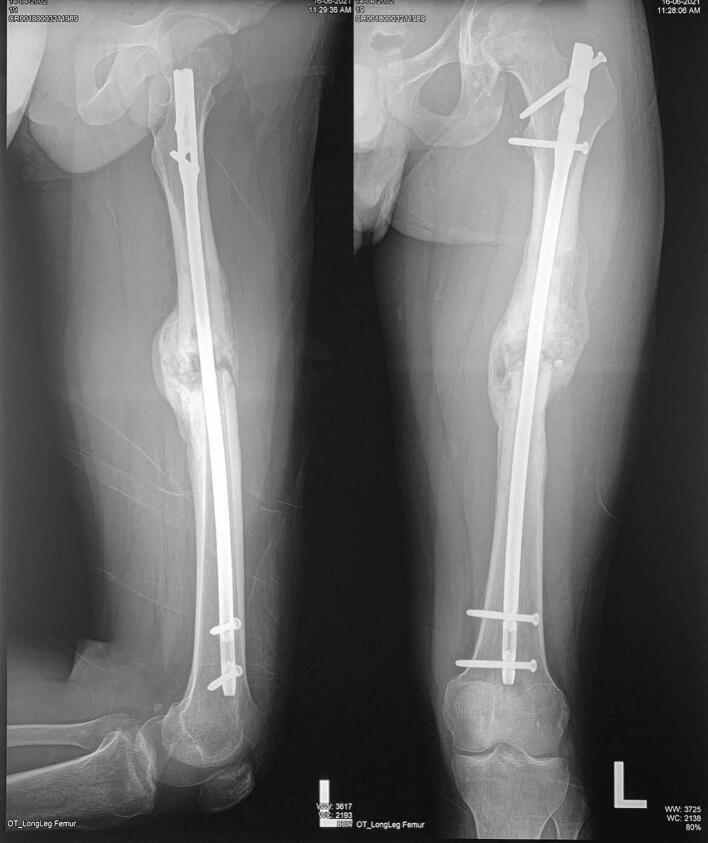
Radiological evaluation after 6 months of revision surgery showing abundant callus formation.

The second patient is a 71 year-old with nonunion of left shaft femur for 2 years. The patient was treated with two-stage surgery: distraction with external fixation and conversion to plate with bone graft application (Fig. 5). The patient was a heavy smoker and had diabetes mellitus type 2. Fig. 6 showed nonunion in the following 6 months and subsequent revision with IM nailing. The patient had decent functional outcome for standing and walking, although having limitation in knee flexion, as shown in Fig. 7. Last x-ray was shown in Fig. 8.

**Fig. 9:**
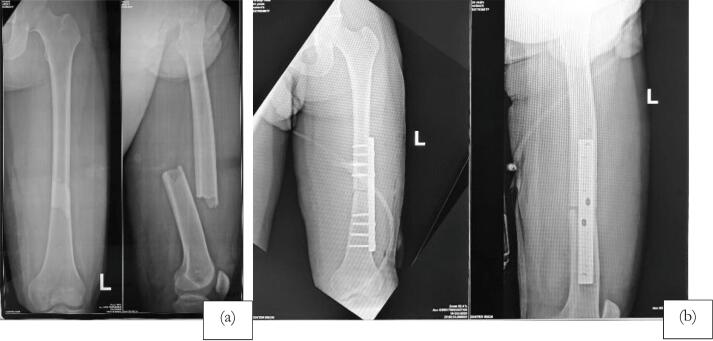
(a) Preoperative X-ray demonstrated fracture of the left femoral shaft. (b) Initial radiological presentation and post-operative x-ray with plate fixation.

**Fig. 10:**
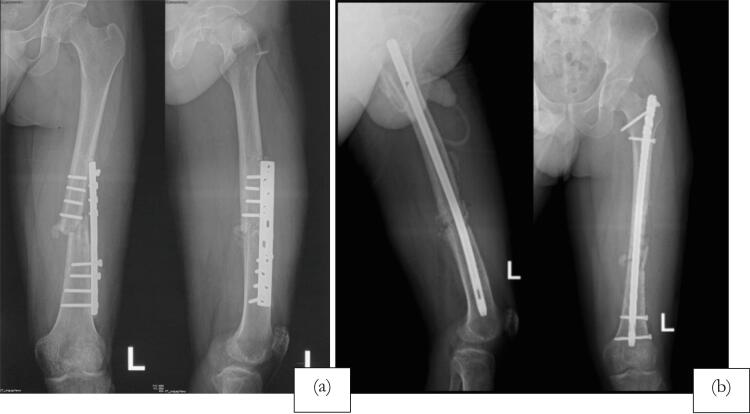
(a) At 3 months after surgery, the patient had implant failure. (b) The patient then underwent revision surgery using intramedullary nailing.

**Fig. 11:**
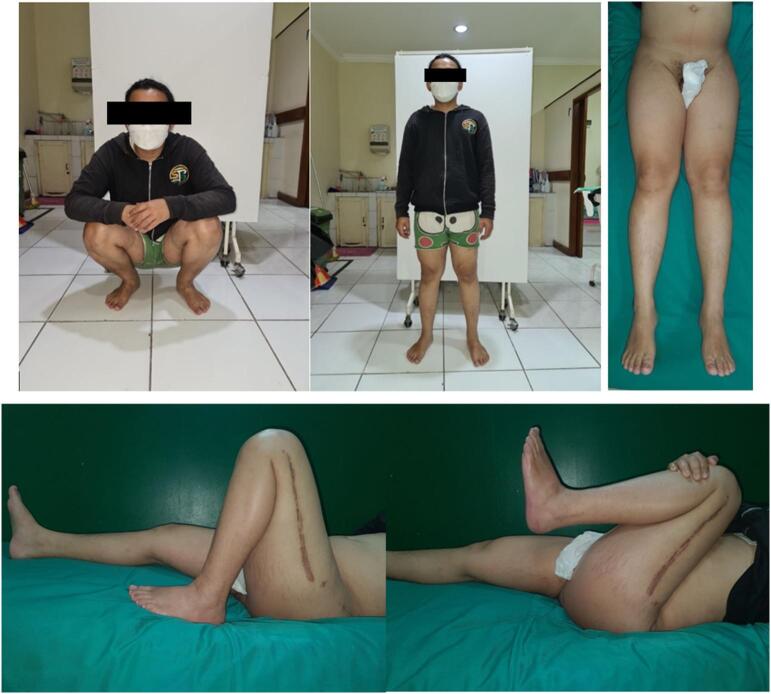
Six months after surgery, the patient had regained full hip and knee range of motion.

**Fig. 12:**
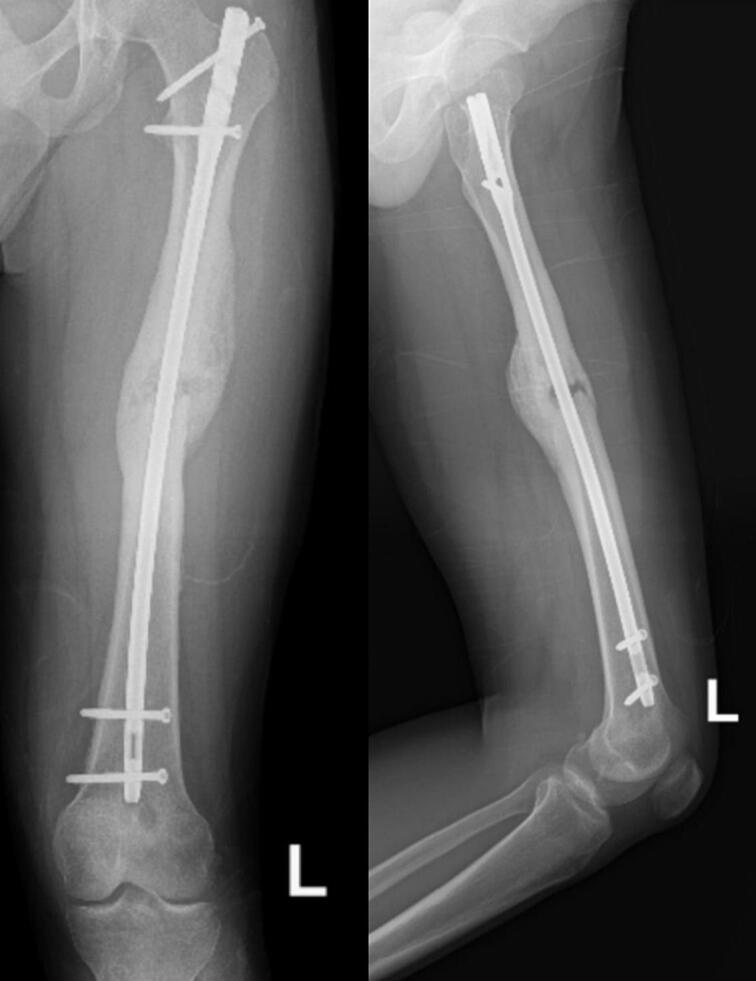
Partial union one year after revision surgery with intramedullary nailing.

The third patient is a-24-year-old male with closed fracture of the left femoral shaft. The patient underwent internal fixation with plating (Fig. 9). After 3 months, the patient came back with implant failure and had revision surgery using intramedullary nailing (Fig. 10). We followed up the patient the patient showed satisfying clinical and radiological outcome (Fig. 11, Fig. 12).

From 3 cases presentation of the patient, the functional outcome was evaluated using Lower Extremity Functional Score (LEFS). The LEFS score of the first patient was 98.8 %. The LEFS score of the second patient was 90 %. The LEFS score of the third patient was 97.5 %. From the evaluation of the x-ray revealed callus formation around the fracture site. We also evaluate the Visual Analogue Score (VAS) of the three patients. The VAS preoperative was 7, 7, and 8 from the three patients, respectively. The VAS 6 months postoperative was reduced into 1, 1, and 2 from the three patients respectively.

## 3 Discussion

Nonunion of the femoral shaft after failed plating does not get much attention nowadays in the developed countries. However, in third world countries, this remains a common problem. Existing evidence recommend nail fixation over other techniques, including plating or external fixation in managing fresh midshaft fractures, and aseptic nonunited fractures. This recommendation is supported by the high union rate.

The management of implant failure in femoral shaft fracture especially if associated with delayed union or nonunion is a dilemma for orthopaedic surgeons. It represents a serious postoperative complication for the patient, associated with longer time of restricted mobility, shortening, deformity, knee joint stiffness and the need for second major surgery, providing osteosynthesis plus bone graft after failed plate removal [8].

Numerous factors are related to implant failure, such as rigidity of internal fixator, early unrestricted weight bearing, second trauma before complete healing of the fracture, concealed infection slowing healing process and poor technique of fixation (too short plate, screws in the fracture site or screws purchasing near cortex only) [8]. In this series, we found that prolonged tobacco use as the risk factor in all patients, while type 2 diabetes was also found in one patient. We predicted that the cause of bony non-union is form the patient's disease that interrupt biological condition and bone healing.

IM nailing is the mainstay of treatment for patients with femoral shaft fractures. This intervention provides support to fractures and aid in union of fractures. Several advantages have been reported in IM nailing, including shorter length of stay, rapid union, and early functional capacity of the limb [11].

In 1970s, IM locking nails for fixation of diaphyseal bone fractures were established as the standard technique in orthopaedic trauma care [12]. The indications of reamed IM nailing in the treatment of femoral nonunions were extended with the introduction of locking techniques, once again with excellent union rates reported for a single nailing procedure [13]. Insertion of IM nailing may preserve anatomical structure in the patients, which leads to better improvement of functional capacity. Nailing also limits soft tissue damage. However, relatively high rate of nonunion has been reported (4.1 % to 12 %) [14]. From this study, we applied IM nail for the three patients and the outcome evaluation was excellent based on the LEFS and VAS.

Our patients presented with plating failure. Seligson et al. [15] previously reported that primary plating has been associated with higher rate of complications of fracture healing (30 %) such as nonunion compared to IM nailing. Furthermore, in patients with failed plate fixation, IM nailing with reaming of medullary canal has shown a union rate up to 96 %. Canadian Orthopaedic Trauma Society also reported that risk of subsequent nonunion was higher in patients with IM without reaming compared to those with reaming in patients with femoral shaft fractures. Therefore, in patients presented with previous plate failure similar to our patients, IM nailing with reaming is recommended [16].

Management of femoral shaft fracture nonunion remains a difficulty in orthopaedic surgery. There are several major postoperative concern for the patient, including implant failures such as broken plate and/or screw, screws loosening or extruding, refracture following plate removal, nonunion, and infection [17], [18], [19]. Other surgical options for treating femoral shaft delayed and nonunion include nail dynamization, nail exchange, plate osteosynthesis, and monolateral external fixation [20], [21]. In this series, we demonstrate that IM nailing may be an effective choice for treating nonunion of femoral shaft fracture.

## 4 Conclusion

Nonunion are common in fractures, including femoral shaft fractures. Conversion from plate and screw to IM nailing may be required to treat non-unions of the femoral shaft fractures.

## Patients consent

Written informed consent was obtained from the patients for publication of this case report and accompanying images. A copy of the written consent is available for review by the Editor-in-Chief of this journal on request.

## Ethical approval

Ethical approval was waived by the authors institution.

## Funding

The authors received no financial support for the research, authorship, and/or publication of this article.

## Guarantor

Ihsan Oesman.

## Research registration number

Not applicable.

## CRediT authorship contribution statement

Ihsan Oesman: study concept, data collection, data interpretation, and writing the paper
Dody Kurniawan: study concept, data collection, data interpretation, and writing the paper
Anissa Feby Canintika: data interpretation, and writing the paper.

## Conflicts of interest

The authors certify that they have no affiliations with or involvement in any organization or entity with any financial interest or non-financial interest in the subject matter or materials discussed in this manuscript.
